# Editorial: The Future of Sport Business

**DOI:** 10.3389/fspor.2021.839520

**Published:** 2022-01-24

**Authors:** Hans Westerbeek, Rochelle Eime, Adam Karg, Veerle de Bosscher

**Affiliations:** ^1^Institute for Health and Sport, Victoria University, Melbourne, VIC, Australia; ^2^School of Science, Psychology and Sport, Federation University, Ballarat, VIC, Australia; ^3^School of Business, Law and Entrepreneurship, Swinburne University of Technology, Hawthorn, VIC, Australia; ^4^Department of Sports and Movement Sciences, Faculty of Physical Education, Vrije Universiteit Brussel (VUB), Brussels, Belgium

**Keywords:** future thinking, sport business insights, disruptive conditions, industry innovation, value creation

There has never been a better time to consider the future of sport business than during a global pandemic that has severely impacted both the community and professional sport communities. These disruptive impacts have been wide ranging, affecting the delivery of sport as well as the financial and social outcomes delivered by sport organizations. As noted by Smith and Westerbeek (2004), “we care about the future because we have to live in it, as will our children and their children. It is important that they have the opportunity to experience and enjoy sport… which requires a healthy global sport industry” (p. xi). The impact of COVID-19 continues to show that there is no returning to “business as usual” and as such, sport organizations (will) have (to) reinvent themselves and their underpinning business models. This is not only to survive, but thrive in continued disruptive conditions. To that end it is important to realize that the business of sport is not limited to the front office of those who run the management and marketing of sport organizations. Value is created (and hence business developed) across the whole of the value chain of sport – on and off the field, from sporting goods manufacturers to athlete management agencies, and by an army of millions of volunteers in community sport. From a number of perspectives, six teams of authors have provided various insights by contributing their work to this Research Topic.

Westerbeek and Eime describe the changing social and policy context in which sport is produced, delivered, and consumed as context and foundation for an integrated framework that incorporates participation in sport with participation in leisure-time physical activity more broadly. In order to position sport in the broader context of leisure-time physical activity, the concept of physical literacy is reviewed and integrated into the theoretical foundations of a new lifespan framework—the Physical Activity and Sport Participation (PASP) framework. The authors express the hope that in recognition of the changing patterns of participation in physical activity and sport across the lifespan, the PASP framework can contribute to coordinated and integrated physical activity and sport policy development, which, in turn, can lead to holistic strategies that tackle the global physical inactivity crisis.

The purpose of the study conducted by Jang et al. was to identify consumer groups through consumer segmentation in the exponentially growing esports industry. The study focused on “game experiences” in the context of esports gameplay consumption. They developed a matrix of esports gameplay based on high/low esports gameplay, viewing esports, and hardware enthusiasm leading to four esports gameplay consumer groups: all-around gamer, conventional player, observer, and recreational gamer. The proposed esports consumers' clustering is an example of research that will contribute to mapping and defining one of the world's fastest growing sport business sectors.

Foster et al. have responded to the growing demand for data analytics talent in (elite) sport organizations. As hiring analytics talent is critical, applicants can command high levels of compensation, in part because they are attractive to companies in many other industries. One of the implications of this broader adoption of analytics across elite sports clubs, will be a greater emphasis on continued innovation across many areas such as player squad assembly, pre-game and within-game strategy, and health and fitness. A further implication is that executive talent from outside of professional sport is increasingly more relevant and valuable to the sport industry. In the near future, the demand (and hence competition) for off-the-field talent in sport business will increasingly intersect with other industries, opening opportunities for cross-pollination and innovation beyond the boundaries of the sport industry.

In their contribution, Jackson and Dawson reflect on the meaning, value and significance of sport. They introduce the challenges facing the global business of sport and describe the privileged position of sport in society, as “sporting exceptionalism.” They then use the example of women's professional sport to illustrate three key steps for an alternative future, while also signaling risks associated with following a hegemonic male model of consumer capitalist sport. They conclude that the more dominant or hegemonic any existing social system is (including sport), the more difficult it is to consider alternative futures. However, there lies tremendous potential value in applying the concept of alternative futures to envisioning a direction for the sport industry. It allows necessary contemplation about new structures, policies, and programmes that will positively transform global sport business.

Chmait and Westerbeek look at how artificial intelligence has transformed the way in which sport is consumed and analyzed. In this perspective paper, the authors present a high-level, non-technical, overview of the machine learning paradigm that motivates its potential for enhancing sports (performance and business) analytics. Founded on a summary of relevant research literature in areas where artificial intelligence and machine learning have been applied to the sport industry, they present some hypothetical scenarios of how artificial intelligence and machine learning could shape the future of sports. They conclude their work with ominous questions. Will artificial intelligence 1 day be shaping the spending behavior of sports fans by exploiting their fan infused emotional vulnerabilities? Or if indeed, artificial intelligence will sacrifice the health of some athletes in favor of the bigger team winning the championship?

In the final article of the Research Topic, Jenkin et al. focus their attention on the rapidly growing, but largely ignored market segment of older adults as sport participants. They investigated how managers in Australian National and State Sporting Organizations perceive sport for older adults. Contextualized in the perspective of organizational change, a framework for marketing to the aging consumer was used to interpret the results. They found that older adults are not a high priority group for most sporting organizations, despite recognition of the benefits of engaging older adults. A lack of age-appropriate programmes was perceived to be a major barrier of engaging older adults. Across all sporting organizations there was broad agreement that increasing participation numbers and engaging the older fan base was important, but most sporting organizations are not (yet) ready to build “age friendly” sporting environments. The study provided insights into why this untapped market is not a priority target, and offers policy makers directions to better engage with this population group.

Across the whole of the value chain, the future of sport business will be as much determined by advancements in artificial intelligence, machine learning and sophisticated (fan and performance) data analytics as it will be through application and sourcing of new business models, tapping new markets, creating alternative revenue streams, management approaches such as alternative futures scenarios, and developing strategic partnerships beyond the sport industry. With a focus on how it impacts the business of sport, in this Research Topic we have covered a number of issues that will strongly influence the future production and consumption of elite and community sport offerings.

## Author Contributions

All authors listed have made a substantial, direct, and intellectual contribution to the work and approved it for publication.

## Conflict of Interest

The authors declare that the research was conducted in the absence of any commercial or financial relationships that could be construed as a potential conflict of interest.

## Publisher's Note

All claims expressed in this article are solely those of the authors and do not necessarily represent those of their affiliated organizations, or those of the publisher, the editors and the reviewers. Any product that may be evaluated in this article, or claim that may be made by its manufacturer, is not guaranteed or endorsed by the publisher.
